# Bite through pain – an experimental pain force-controlled study on EMG-activity of the masseter muscle

**DOI:** 10.1007/s00784-026-06885-w

**Published:** 2026-04-25

**Authors:** Johannes P. van Dijk, Sophia Terebesi, Hans J. Schindler, Peter Svensson, Ulrike Eiglsperger, Bernd G. Lapatki, Nikolaos Nikitas Giannakopoulos

**Affiliations:** 1 Center of Dentistry, Department of Orthodontics, Ulm University, Albert-Einstein-Allee 11, 89081 Ulm, Germany; 2 Department of Clinical Physics, Epilepsy and Sleep Center Kempenhaeghe, Heeze, The Netherlands; 3 Department of signal processing systems, Eindhoven University of Technology, Eindhoven, The Netherlands; 4 Department of Prosthodontics, University of Würzburg, Josef-Schneider-Straße 2, 97080 Würzburg, Germany; 5 Faculty of Dentistry, National University of Singapore, National University Centre for Oral Health, 9 Lower Kent Ridge Road, 119085 Singapore, Singapore; 6 Department of Prosthodontics, National and Kapodistrian University of Athens, 2 Thivon street, Athens, 11527 Greece

**Keywords:** Masseter, Surface electromyography, EMG, Orofacial pain, Healthy participants, Hypertonic saline

## Abstract

**Objectives:**

Aim of this high-density surface electromyography (EMG) study was to evaluate the effect of experimentally-induced pain in the masseter on the performance of a constant vertical bite-force.

**Material and methods:**

Twenty healthy participants performed two ramp-and-hold contractions at baseline and after an injection of either hypertonic saline (HS) or, as control, isotonic saline (IS) based on random assignment. The measurement was repeated after 15 min. with IS or HS, respectively. The target level of vertical bite-force was 15% of maximum voluntary contraction registered using an intra-oral device.

**Results:**

The average root-mean-square (RMS) values showed significantly lower (*p*<0.001) EMG activity for all selected monopolar (14.9%) and bipolar (17.1%) derivations for the HS condition despite unchanged mean force. Bipolar EMG RMS values for the contralateral masseter did not change significantly. The centre of mass obtained from the interpolated monopolar RMS amplitude map showed a mean shift away from the injection site of 0.85mm (*p*<0.001).

**Conclusions:**

Experimental pain induced a general reduction of RMS EMG amplitude of the ipsilateral masseter and a small but significant shift away from the site of injection.

**Clinical relevance:**

The masticatory motor system seems to possess a unique adaptability to produce a constant force despite the presence of experimentally induced pain. This could represent a feature of the high resilience and adaptability of the stomatognathic system in acute painful conditions.

## Introduction

It is a common experience that pain interferes with movement but exactly how motor performance is changed has been a source of much debate and controversy over time. Accordingly, different theories have tried to describe the effect of pain on muscle activity and performance. Starting with the Vicious Cycle Theory [1, 2] and later the Pain Adaptation Model [3], muscles have mostly been regarded as a single unit of force production changing their function in response to pain depending on their primary role as agonists or antagonists. More recently published models, such as the integrated Pain Adaptation Model [4] and the Theory of Motor Adaptation to Pain [5], propose a redistribution of activity between regions within a muscle or between muscles in an individual- and task-specific way. A recent model called “Theory of Pain Sensory-Motor Interactions” highlights the potential for both adaptive and maladaptive changes in motor function dependent on the individual mosaic of genetic, epigenetic, psychological and social factors and their interactions [6]. The aforementioned models coexist, applying at different levels of analysis (muscle, motor unit, CNS) and are best understood as complementary rather than hierarchical.

Evidence suggests that the interrelations between muscle activity and pain are actually more complex even at the level of the motor units (MUs) [5, 7]. Previous studies have shown that experimentally-induced acute pain in the masseter will lead to a reorganisation of MU activity throughout the muscle [8, 9] and the pattern of reorganization may vary in different muscle regions [9]. These findings were established using intramuscular electrodes in the masseter and force-controlled vertical biting tasks, carried out before and after infusion of either hypertonic saline (HS) or (as control) isotonic saline (IS) through a catheter.

Intramuscular injections of HS are considered suitable for inducing acute experimental muscle pain and simulating chronic local myalgia, and is a well‑established, low‑risk method [10, 11]. Moreover, this experimental model allows for standardisation of pain duration, as well as the induction of pain in a restricted muscle region [12, 13]. It provides a unique opportunity to observe the specific changes in motor control and MU reorganisation directly associated with the painful stimulus. In contrast, in patients with clinical pain, this transition from a situation without pain to a painful situation is difficult to investigate.

Among electromyographic (EMG) techniques, intramuscular electrodes have the advantage of being closer to the recorded MUs. A downside, nevertheless, may be their invasiveness and, consequently, the potential impact on muscle function and pain perception in participants. This may be of particular importance in the context of studies on pain. Moreover, if pain leads to a reorganisation of MU activity that causes spatial changes without affecting the motor performance, this phenomenon may be better investigated with high-density surface electrodes covering the entire muscle. One previous study that has employed a combination of surface and intramuscular electrodes has found a significant decrease in EMG activity in all recorded masseter regions [14], however, biting tasks were investigated in this study without objective force control.

The aim of this study was to assess, by means of high-density surface EMG, the effect of experimentally-induced pain in the masseter muscle on the generation of a constant vertical bite-fore in healthy participants. We hypothesized that pain does not alter the motor performance of the masticatory system, despite (globally or regionally) changing the EMG activity registered from the masseter.

## Materials and methods

### Participants

Twenty healthy participants of similar age (nine women, eleven men; mean age 27.2 ± 4.7 years) were enrolled in this investigation. All participants were naturally dentate, without pain or dysfunction as assessed by the Diagnostic Criteria for Temporomandibular Disorders, DC/TMD [15]). The study was approved by the Ethics Committee of the University Medical Faculty, Würzburg (no. 100/17-mk). All participants provided written informed consent to the experiments. The experimental procedures were conducted in accordance with the World Medical Associations’ Declaration of Helsinki (version from October 2013).

### Force recording

Dental impressions were made for individualization of the intraoral force transducer. Non-occluding acrylic splits (3.0 mm thickness; Erkodur; Erkodent, Pfalzgrafenweiler, Germany) were fabricated for the upper and lower dental arches. The mandibular device was prepared with self-curing resin for insertion of a “bearing pin,” equipped with strain gauges for measurement of horizontal forces [16]. This sensor provided visual feedback for horizontal mandibular displacement. On the maxillary device, an acrylic surface with a fitting indent was established. Its function was to activate the bearing pin, in case of movement of the mandible away from the predetermined position established by the intercuspation position of the participant. Measurement of the bite-force magnitude was achieved by use of a hydrostatic system described in detail elsewhere [16]. Briefly, bite forces on the pads resulted in increased hydrostatic pressure within the liquid. The forces were measured by use of a specific sensor device (Althen, Kelkheim, Germany). Stable positioning of the pads was achieved by stabilizing them on pad holders attached to the intraoral devices. Liquid level within the pad was kept stable by use of a small pump integrated into the hydrostatic system.

### Experimental procedure

The experimental pain protocol included intramuscular injection of 0.25 ml 5% HS and same amount of IS in a randomized order into the palpated belly of right masseter muscle. For this purpose, a disposable 24-gauge (19-mm length) needle integrated in a custom-made infusion pump was inserted about 20 mm through a perforation in the surface electrode grid. The bolus infusion of 0.25 ml 5% HS was infused continuously over 20 s at an infusion rate of approximately 0.0125 ml/sec. Pain intensity was assessed by the participants on a 100-mm numeric rating scale (NRS; 0 mm, no pain at all; 100 mm, the worst possible pain) ) at the following time points: prior to the injection, after commencement of infusion until pain reporting reached its peak, and after each ramp-and-hold task. All participants were randomly assigned to one of two groups according to the order of the experimental pain induction. In one group, the first injection was the HS, and the other was injected first with the IS solution. Participants were blinded to the order of injections, but because the second injection clearly differed from the first, they could practically infer which solution would follow. After the HS protocol measurements, participants were asked to complete the Pain Catastrophizing Scale (PCS) based on the pain they just experienced. The PCS is a psychological tool used to assess the extent of catastrophic thinking related to pain. It consists of 13 items; each rated on a 5-point scale from 0 (“not at all”) to 4 (“all the time”). The total score can range from a minimum of 0 to a maximum of 52, with higher scores indicating greater levels of pain catastrophizing [17].

The EMG activity of the right masseter was measured with a high-density surface EMG electrode grid consisting of 256 regularly spaced Ag-AgCl contacts 1 mm in diameter and an inter-electrode distance of 3 mm. The grid was placed in a standardised way as previously described [18]. Briefly, after palpating the muscle belly and marking it on the skin, a line was drawn between the jaw angle and the lateral eye corner. This line was centred between two specific electrode columns. The lower border was aligned to the palpated lower mandibular border defining the vertical grid position. A separate reference electrode was placed on the nose for monopolar derivation. On the contra-lateral side, a conventional bipolar surface electrode was placed over the masseter belly with an inter-electrode distance of 2 cm. The EMG data was acquired by a 256-channel amplifier (ActiveTwo; BioSemi, Amsterdam, The Netherlands) sampled at 4096 Hz with a resolution of 31.25 nV.

**Fig. 1:**
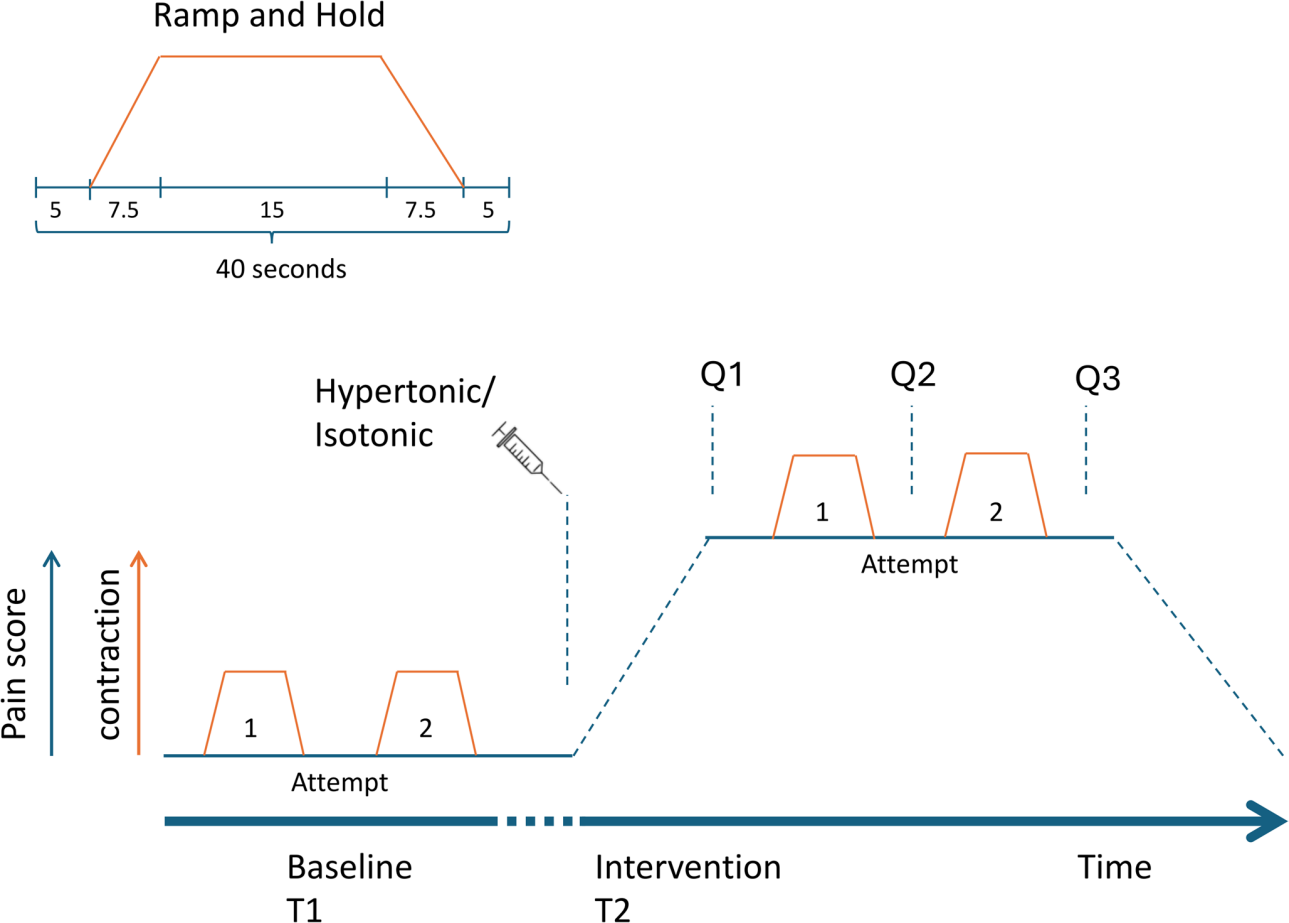
Experimental task consisted of four ramp-and-hold contractions (RHCs) at a level of 15% MVC during the holding phase. Each RHC was repeated twice (attempt 1 and 2) at baseline (T1) and after the intervention (T2) for analyzing reproducibility. During the contractions, the participants received visual feedback of the exerted force in time and the targeted force level on a display. The intervention consisted of injection of either hypertonic (HS) or isotonic saline (IS) into the right masseter. At Q1-Q3 the participants were asked to indicate the perceived pain on a visual analogue rating scale, and if the pain changed during the contraction. After the pain completely disappeared, a pause of 15 min. was introduced before the second intervention type started. The sequence of the two intervention types was randomized

Participants were seated on a chair and performed two ramp and hold contraction (RHC) vertical bite force tasks separated by a rest period of 10 s in between (Fig. 1). RHC were performed at baseline, during maximum pain and after pain reporting had returned to 0. In a preceding session, the participants were familiarized with the task to follow the ramp and hold pattern as precise as possible within the given time frame of 30 s.

In the recording session, the intraoral experimental devices were bonded to the teeth using cyanoacrylate adhesive (multi-purpose super Glue; Renfert, Hilzingen, Germany). Then, the surface EMG electrodes were placed. As the pads for force recordings cannot withstand extreme bite forces, the individual bite force for the experiments was determined by means of three repetitions of maximum bite forces on cotton rolls, while monopolar EMG was recorded for definition of 100% MVC. After replacing the cotton rolls by the force pads, the participant was provided with EMG amplitude feedback via a monitor and instructed to perform an EMG amplitude corresponding to a 15% MVC contraction. The force recorded on the pads was noted and used as maximum force level in the ramp and hold contraction tasks. To avoid that the participants deviate from the pure vertical force direction, they were asked to establish the least horizontal force possible, controlled via use of the feedback monitor. In this way, the participants could precisely adjust both jaw position while biting on the pads simultaneously. The position of the lower jaw had to be maintained stable for 30 seconds, 7.5 seconds for gradually achieving the predetermined force level on the ramp, 15 seconds holding this force level and 7.5 seconds progressively decreasing the force on the ramp. The RHC procedure was repeated twice with a 10 second rest between the contractions, for each experimental condition. The measurements took place at baseline (T1, before any injection) and shortly after injection of HS (T2, at the pain peak) or IS (T2’). The participants were asked to report their pain level on the NRS at the following time points: as soon as the pain level was stable 10 s after the injection, after the first and the second contraction, and when no pain was reported. Moreover, after each contraction, the participants were asked about the pain perception (stable, increased, decreased) during the ramps. To prevent muscle fatigue, a break of 15 min after reaching pain-free state was implemented between replicated measurements, which also served as wash-out period.

### Data analysis

**Fig. 2:**
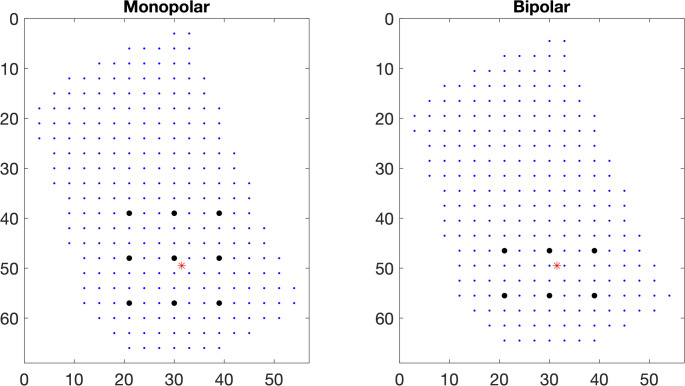
Representation of the high-density surface EMG electrode with 256 contact points and an inter-electrode distance of 3 mm. For the monopolar plot each blue dot represents an electrode contact. The red star represents the injection site for this participant. The black dots represent the position of the signals used for further analysis

To determine how pain effects the ability to follow a challenging task as the RHC task the participant had to perform, force profiles per RHC were analysed by determining the root mean squared error (RMSE) between the target line the participant had to follow and the force sensor feedback line. Secondly, a stable force period of 2 s was determined manually for each RHC during the hold phase. Analog to a previous study [14], after high-pass filtering (10 Hz, 2nd order Butterworth), we determined the bipolar RMS amplitude and median frequencies for 3 bipolar derivations located around the injection site, i.e. directly above this site, 9 mm posteriorly, and 9 mm anteriorly, with an inter-electrode distance of 9 mm (Fig. 2). Moreover, monopolar RMS amplitudes and median frequencies (reference to the nose) were determined on 9 locations evenly spread around the injection site with a 9 mm interelectrode distance.

Based on a previous study [19], the modified entropy is calculated over the entire electrode grid for each contraction to determine a possible change in amplitude distribution over the high-density electrode. Finally, to determine a possible shift of EMG activity away from the injection site, the centre of mass (CoM) was calculated over the 8 times interpolated electrode grid for the area in which the monopolar RMS amplitude was 50% above the maximum RMS value. This way the centre of mass indicates the location where the RMS amplitude is high but is less sensitive to local maxima. The Euclidian distance between the injection site and the CoM was used to denote a possible shift in activity.

### Statistics

Linear mixed effect (LME) models were used to analyse the effect of IS versus HS injection for the RMSE (entire force profile), and mean force level, RMS amplitudes on different electrodes, shift in CoM and modified entropy (for the 2 second table hold phase). For each model a check for normality of residuals and heteroscedasticity was performed. Note that the sequence of HS and IS injections was also included into the models to determine a possible effect of suboptimal randomization as participants were blinded practically only for the first injection. All participants received the same sensory cues and interaction for each recording. For the median frequency a similar LME model was used but here the output variable was the mean frequency. Differences were considered significant if *p* < 0.05. Marginal (Rm) and conditional (Rc) R^2^ values according to Nakagawa and Schielzeth are provided for the LME model outcomes [20].

## Results

### Pain reports

After the injection of HS (Fig. 1 Q1), at the peak of the perceived pain, most participants (*N* = 13) reported moderate pain levels (NRS 4–6), six participants indicated severe pain (NRS > 7) and one participant reported mild pain (NRS < 3). The average pain level was 5.9 (SD = 1.6, range: 3–9). After the first RHC was performed (Q2), the average NRS decreased to 5.6 (SD = 2.0, range: 3–9) and further decreased to 5.3 (SD = 2.3, range: 1–9) after the second RHC (Q3). For the IS condition almost all participants indicated a maximum pain level of 0 at all time points (*N* = 16), while two participants indicated a pain level of 1 and one participant indicated a pain level of 2. Mean and SD pain scores before the first and second contraction and after the second contraction were 0.2 (0.5), 0.15 (0.4) and 0.05 (0.2) respectively.

During the first RHC, most participants (*N* = 13) reported an increase in pain for the HS condition while they remained unchanged for 5 and decreased for 2. For 14 participants pain levels increased during the second RHC while 6 participants reported unchanged pain levels.

The PCS questionnaire was obtained from all participants except 1 due to an administrative error. The PCS was quite variable among participants with mean of 12.2 ranging from 2 to 27. We found no significant correlation between PCS and NRS values obtained prior to the first and second RHC (Pearson correlation; *r* = 0.24, *p* = 0.32 and *r* = 0.32, *p* = 0.18 respectively).

### Bite force

**Fig. 3:**
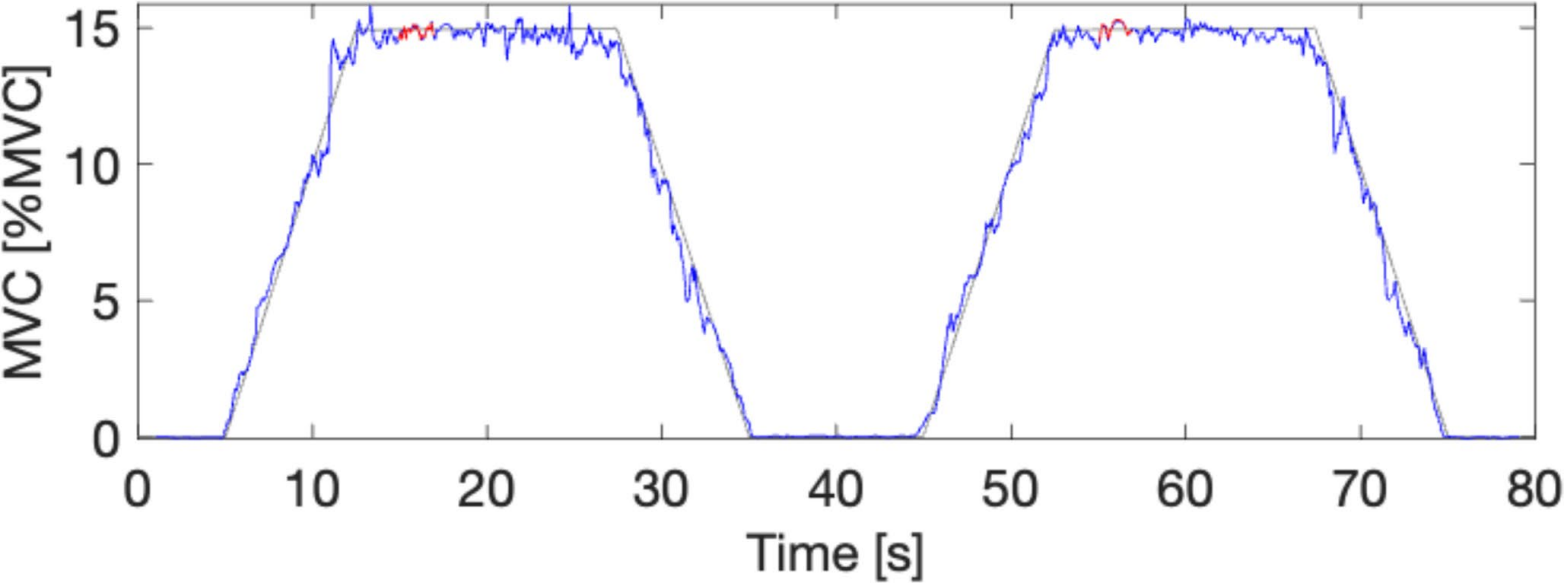
Typical example of a RHC force profile. The thin grey line indicates the target line for the participant, while the blue line represents the force level (in % of MVC) exerted by the participant on the force transducer. The red selected (within the rectangle) part reflected the most stable interval during the 15-sec. holding phase and was used for further analysis

Over all contractions, the mean force level determined for the 2 stable seconds was held almost perfectly at the targeted level of 15% MVC (see Fig. 3 for a typical example). For one participant the mean force target after the first RHC attempt was reduced to 10% MVC due to a technical issue. Therefore, this participant was removed from further analyses. The mean force level (for 19 participants) was 14.8% MVC (SD: 0.3%) and 14.7% (SD: 0.3%) during IS and HS conditions, respectively. Statistical analysis using an LME (*N* = 19) showed that there was no significant difference in mean force (*p* = 0.072, Rm = 0.04, Rc = 0.15) for IS and HS conditions. The LME model, included “intervention type” and “attempt” (first or second RHC) as fixed effects and “participant” as random effect. Moreover, the model showed “attempt” was not a significant factor (*P* = 0.79). Force variability expressed as the standard deviation of the force level over the same 2 s of data (as was used for the mean force) was also not significant either between the HS and IS (*p* = 0.7, Rm = 0.02, Rc = 0.2) conditions, indicating no difference was found in the ability of the participants to maintain a stable force during pain.

We also calculated an LME model to determine whether the RMSE for the entire RHC profile differed between “Intervention type”, “Attempt” and “Mean_force” (fixed effects), using “Participant” as random effect. The model revealed no significant effects for any of the three fixed effects for neither values at baseline (T1, *p* = 0.96, *p* = 0.26, *p* = 0.051 respectively, Rm = 0.05, Rc = 0.62), nor after intervention (T2, *p* = 0.33, *p* = 0.78, *p* = 0.11 respectively, Rm = 0.06, Rc = 0.18).

### EMG analysis

We analysed EMG RMS values for the nine selected monopolar EMG electrode contacts on the ipsilateral masseter and the single bipolar electrode on the belly of the contralateral masseter. In the corresponding LME model, baseline EMG RMS amplitude values, “Attempt”, “Mean_force”, and “Sequence” were incorporated into the model as a fixed effects, and “Participant” as random effect.

For each nine ipsilateral electrode locations, monopolar RMS EMG amplitudes showed significant (*p* < 0.001) lower values for the (painful) HS condition. The estimated effect size on average over all nine electrodes was − 0.014 (range − 0.009 to -0.019) corresponding to a mean percentual amplitude change of 14.9% (Table 1). The bipolar EMG RMS amplitude on the contralateral masseter was not significantly different between IS and HS injections (Table 2).

**Table 1:** Outcome of linear mixed effect (LME) models with monopolar root mean square EMG amplitude (RMS) as output variable for all 9 electrode locations (Figure 2). Baseline EMG RMS amplitude values, “Attempt”, “Mean_force”, and “Sequence” were incorporated into the model as a fixed effects, and “Participant” as random effect. The table shows exact p-values and the mean change for each of the electrode locations as well as the marginal (Rm) and conditional (Rc) R^2^ values

Electrode	Estimate	*p*-value	Rm	Rc	RMS	mean Change
1	-0.018	1.52E-05	0.40	0.74	0.108	-17.0%
2	-0.014	9.37E-05	0.58	0.74	0.093	-15.0%
3	-0.017	2.18E-04	0.53	0.73	0.124	-13.9%
4	-0.011	5.35E-05	0.81	0.81	0.066	-15.9%
5	-0.019	2.04E-05	0.61	0.70	0.105	-17.9%
6	-0.009	6.32E-04	0.75	0.78	0.067	-13.0%
7	-0.012	9.73E-04	0.58	0.72	0.100	-12.4%
8	-0.009	2.25E-05	0.86	0.86	0.062	-15.3%
9	-0.019	3.36E-04	0.52	0.70	0.139	-13.5%
mean	-0.014	2.63E-04	0.63	0.75	0.096	-14.9%
min	-0.019	1.52E-05	0.40	0.70	0.062	-17.9%
max	-0.009	9.73E-04	0.86	0.86	0.139	-12.4%

**Table 2:** Outcome of linear mixed effect (LME) models with bipolar root mean square EMG amplitude (RMS) as output variable for all 3 electrode locations (Figure 2). baseline EMG RMS amplitude values, “Attempt”, “Mean_force”, and “Sequence” were incorporated into the model as a fixed effects, and “Participant” as random effect. The table shows the exact p-values and the mean change for each of the electrode locations as well as the marginal (Rm) and conditional (Rc) R^2^ values

Electrode	Estimate	*p*-value	Rm	Rc	RMS	mean Change
1	-0.018	8.67E-06	0.49	0.73	0.096	-18.4%
2	-0.014	4.94E-05	0.57	0.72	0.090	-16.1%
3	-0.014	9.22E-06	0.77	0.79	0.086	-16.8%
mean	-0.015	2.24E-05	0.61	0.75	0.090	-17.1%
min	-0.018	8.67E-06	0.49	0.72	0.086	-18.4%
max	-0.014	4.94E-05	0.77	0.79	0.096	-16.1%

We found no significant interrelation between the decrease in EMG amplitude and the pain scores reported before the first RHC (*p* = 0.14) or the second RHC (*p* = 0.08, Spearman Correlation).

An LME model was also calculated for the median EMG frequency using the same variables as those for the EMG amplitude. We found no consistent effect over the 9 monopolar or 3 bipolar electrodes on the ipsilateral masseter (*p* > 0.05). None of the other fixed effects showed to be significant either.

### Centre of mass (CoM)

**Fig. 4:**
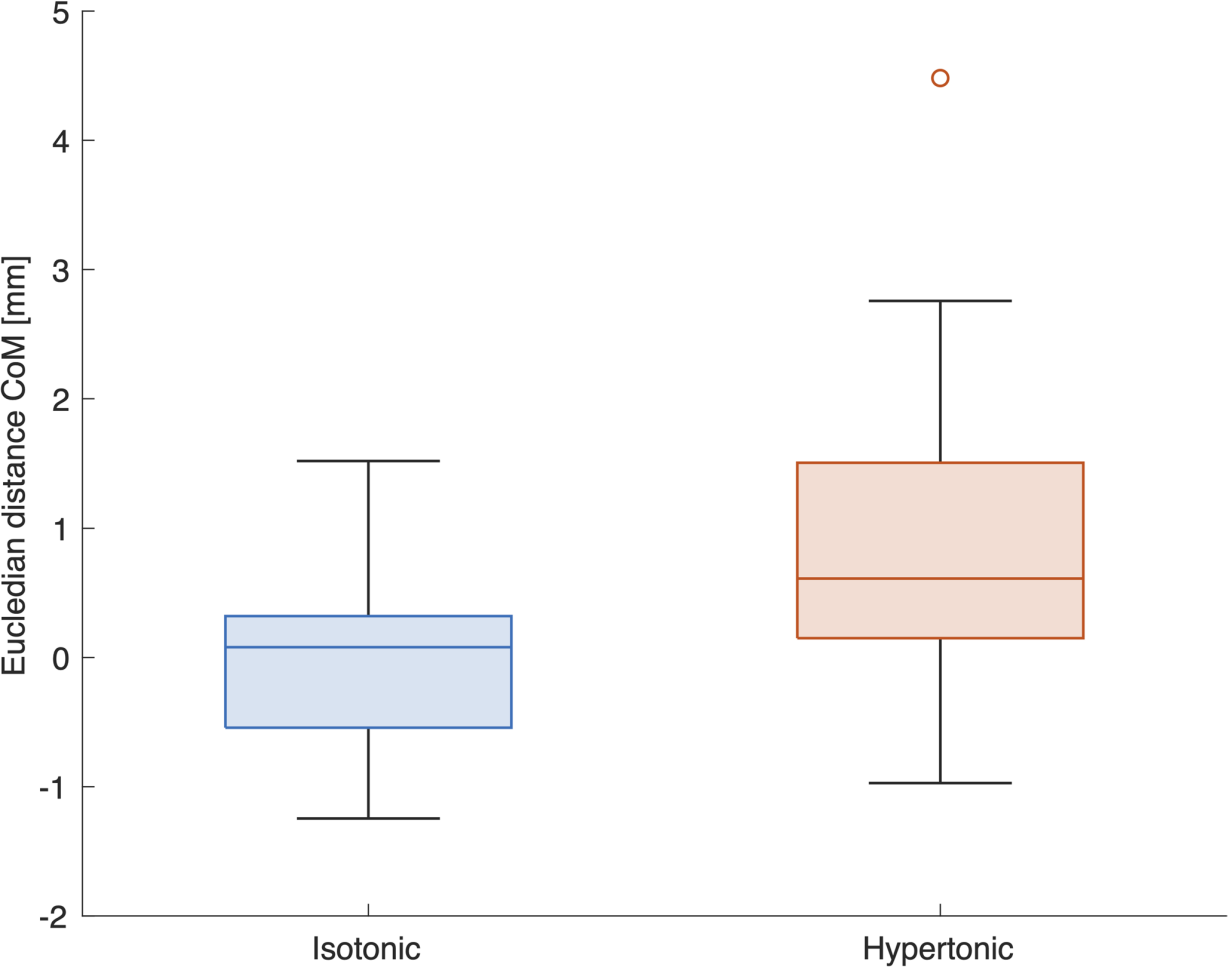
Boxplot for Euclidian distance (in mm) between the Centre of Mass (CoM) of the interpolated monopolar interference EMG amplitude maps and the injection site for both, the isotonic (IS) and hypertonic saline (HS) conditions. The linear mixed effect model (LME) revealed a statistically significant (*p* < 0.001, Rm = 0.957, Rc = 0.963) difference of 0.85 mm between the distances for the two conditions

To determine if an IS or HS injection might cause a shift in the activity distal from the injection site, per participant the Euclidean distance between the CoM and the injection site was determined at baseline and during the second RHC. Distances varied substantially between subjects with minimal value of 2.60 mm and a maximum of 24.12 mm over both conditions. The baseline value was subtracted to obtain the Euclidean distance change per participant for the IS and HS conditions which are shown in Fig. 4. An LME model including “Baseline distance”, “Attempt” and “Intervention type” as fixed effects showed a small, but significant shift of the CoM by 0.85 mm (95% confidence interval [0.52 1.17], *p* < 0.001) away from the injection site for the HS condition, compared to IS. Baseline distance was also highly significant, effect size 0.89 mm (95% confidence interval [0.84 0.95], *p* < 0.001). Attempt was not a significant effect in the model (Rm = 0.957, Rc = 0.963).

### Modified entropy

An LME for the modified entropy values obtained over the entire electrode grid, including “Baseline entropy value”, “Attempt”, “Intervention type” and “Sequence” as fixed effects showed no significant effect of intervention both for monopolar and bipolar derivations (*p* = 0.198, Rm = 0.12, Rc = 0.95, *p* = 0.114, Rm = 0.02, Rc = 0.83 respectively).

## Discussion

The main finding of this experimental study was that the experimental induction of (moderate to severe) pain at a specific region of the masseter caused a global effect on the ipsilateral masseter, although, the motor performance of the masticatory system, in terms of the level and temporal variability of a constant vertical bite force task was not compromised. More specifically, for all EMG recording sites, amplitudes decreased significantly (for the bipolar montages on average by 17%) after HS injection compared to the control IS injection, and the CoM for the RMS maps shifted slightly away from the injection site. Specific features of our experimental study were, that a feedback-controlled RHC task with a well-controlled pure vertical force direction, and application of a high-density surface EMG electrode grid which allowed to evaluate the topographical amplitude changes in more detail when compared to surface EMG studies with a few conventional electrodes. The RHC tasks were performed twice both, prior and after the interventions (attempts 1 and 2), to determine their reproducibility. In any of the analysis neither the variable “Attempt” nor the sequence of HS and IS injections were a significant factor. Moreover, also bipolar EMG on the contralateral side did not show any differences between experimental conditions. These findings may be considered as proof that the effect of EMG change was highly reproducible and that the incomplete blinding of the participants had no effect on the results.

Intramuscular HS infusion is a well-established experimental model for evaluating the effects of acute muscle pain in healthy participants [21]. As a potent nociceptive stimulus, particularly for type IV fibers compared to type III fibers [22, 23], HS serves as also an appropriate model for studying the interaction between pain and motor control [24, 25]. Previous studies have employed this acute pain model, for simulating uni- or bilateral masseter pain [13, 14]. Although HS‑induced experimental pain reliably elicits deep, localized muscle pain, this model has limitations. Acute experimental pain may not reproduce some features of clinical or persistent pain conditions, such as central sensitisation, long‑term cortical plasticity, or altered descending modulation. Consequently, the present findings should be interpreted with caution in this respect. However, the wide range of PCS scores in our and other studies, and the moderate to severe pain reports may indicate a clinically meaningful representation of an acute, deep nociceptive input on jaw motor behavior. Although pain scores differed substantially between participants, we found no evidence for a direct relation between pain scores and EMG amplitude change.

In contrast to our findings, multiple studies have reported no reduction of EMG amplitudes after experimentally induced pain [26–31]. In two studies by Sohn et al. capsaicin-induced experimental pain was used in the masseter with no effect on the RMS value of surface EMG during isometric contraction at 5–15% MVC [27, 28]. In a study by Michelotti et al. a slight increase in EMG activity was reported at 25%MVC clenching task [30]. The latter study reported pain and fatigue increases during the clenching task, which may explain the reduced median frequency in that study. In another study, at 5% MVC, that is noticeably lower than our 15% MVC, there was a significant increase of RMS of the contralateral masseter after HS infusion and no effect on the ipsilateral side [31]. However, the same study reported a decrease of EMG activity on the ipsilateral side for contractions at 50% and 100% MVC. The study by Sandoval et al. showed no detectable differences in EMG activity from the left and right masseter and anterior temporalis muscles [26]. We speculate that possible reasons for the finding a decreased EMG activity on the ipsilateral side in our experiments were the refined methodology for force and EMG recordings and the highly reproducible experimental tasks.

Our results partially agree with those of Türp et al. where the HS solution induced a significant decrease in EMG activity in all recorded muscle regions ipsilateral to the side of mastication [14]. However, in contrast to the latter study we found no effect in EMG amplitude for the control (IS) condition. A likely cause for this difference might again be ascribed to the distinct experimental tasks, i.e. a unilateral mastication versus a RHC task at a submaximal force level. In contrast to our findings, Castroflorio et al. found that masseter RMS values were not significantly decreased for low contraction force levels (20 and 40% MVC) [32]. However, they found a bilaterally effect for higher force levels (60 and 80% MVC) following painful administration of intramuscular glutamate. In our results, however, the contralateral masseter side did not show any change. One reason for this disagreement might be the use of glutamate, which might affect the masseter muscle differently. Furthermore, the lack of change at low contraction levels in their study might have been due to the relatively small sample size (only 9 participants). The fact that we found no change on the contralateral side may also be explained by the relatively low contraction level at which we performed the experiment. In the study by Türp et al., the relative activity reduction differed within the three regions: anterior, intermediate and posterior [14]. In contrast, our findings showed that EMG amplitude was reduced evenly over the entire muscle corresponding with the finding of Catroflorio et al. [32]. Although the activity showed to be evenly reduced over the muscle, a very small (0.85 mm) but statistically significant (*P* < 0.001) shift of the centre of mass of the RMS EMG activity was found away from the injection site. This shift in CoM might be explained by subtle changes in MU recruitment or derecruitment and, hence, could be interpreted as agreement with the Theory of Motor Adaptation to Pain at a very localised level [5].

In this study, experimental pain and the subsequent decrease of EMG activity of the ipsilateral masseter did not affect the absolute mean force nor did it affect the stability of keeping the force level for this RHC task. Moreover, there was no difference between the entire RHC when looking at the RMSE for the entire RHC profiles. The former agrees with another experimental setting, where the injection of 0.2 ml of monosodium glutamate into the masseter reduced the variability of hold force but not of split force, compared with IS and generally did not show any robust effects on the hold and split forces [29]. Regarding the IS injection, our results agree in general with those of Svensson et al. who found the mean EMG activity of all the jaw-closing muscles to be significantly decreased in the agonist phase during bilaterally painful mastication [33]. This shift of balance between synergistic and antagonistic activity might explain how force was maintained in our study despite the reduction in EMG. As we did not obtain EMG from other agonist muscles (e.g. temporalis and medial pterygoid), however, it is not possible to verify this hypothesis.

At the MU level, in conditions requiring a constant force from the masticatory muscles, as in our experiment, there are indications that the firing rate of the active individual MUs decreases without changing the recruitment threshold of the low- and moderate-threshold MUs [27]. Moreover, a significant increase in the amplitude of the twitch force in low-threshold masseter MUs during painful, gentle biting has been shown [28]. In this context, we did not find a change in the median frequency that could indicate an effect of the MU firing rates. However, it must be noted that median frequency may not represent the subtle changes in firing rates found at the MU level. Median frequency may also reflect fatigue, specifically during long contractions, although we would not expect any fatigue effect in our study due to the short contractions at the relatively low contraction level.

According to the recently introduced Theory of Pain-Sensorimotor Interactions (TOPSMI), sensorimotor neuronal outputs and associated MU activation patterns are influenced by a “favourable” or “unfavourable” mix of biopsychosocial factors and their interaction with adaptive glioplasticity and neuroplasticity in nociceptive, modulatory, and sensorimotor circuits [6]. This may involve increased firing rates and recruitments of some MUs and may also be associated with derecruitment and decreased firing rates of other MUs according to a biomechanical advantage and the metabolic cost. The strategies that result from adaptive sensorimotor neuronal outputs are associated with the adoption of MU activation patterns that could lead to pain minimization and homeostasis, whereas those from maladaptive sensorimotor neuronal outputs might result in no improvement and could result in worsening of pain or even the development of new pain. Our study and previous investigations provide evidence that pain affects the recruitment strategy locally within the masseter muscle. As our results on the contralateral side remained unchanged, this indicates that the observed effect is localised and not primarily driven by centralized or bilateral changes under the acute pain conditions – at least in low isometric contraction levels as that was investigated in this study. It may also be concluded that, in accordance with some previous studies, our results may indicate the ability of the stomatognathic system to adapt to acute pain conditions. However, it may be questioned whether the adaptations observed under experimental pain conditions are akin to that might result in a maladaptive pathway. Moreover, the masseter musculature is highly complex and differs from other muscles making generalisations speculative. Further research is necessary, particularly focusing on patients exhibiting early symptoms of TMD, to address this question.

Some limitations must be discussed as well. Our experiment was performed in a homogenous group of 20 young healthy participants which limits its generalizability towards a broad population. Second, as already mentioned, the experimental procedure used is only valid for the acute pain model and may not translate to chronic pain conditions. Furthermore, the investigated 15%-MVC level was determined based on the EMG during a maximum bite force on cotton rolls; consequently, as EMG amplitude is not completely linearly related to force, the exact % MVC might be somewhat higher compared to a direct MVC standardization via a force transducer. This might have caused some extra variability between participants. However, it is noted that because of the within subject design the effect on the results is expected to be negligible. Another limitation is related to the tasks, as RHC tasks with vertical force direction may challenge the motoneurons of the masseter’s neuromuscular system in a different fashion than during the semi-automatic mastication or other “common” or “automatic” tasks. The advantage of this task, however, is that it can be controlled very precisely. Such precision might actually have been required to identify the relatively small effect caused by the applied experimental pain model applied in our study.

Overall, the present study demonstrated that experimental pain induced a general reduction of the activity of the ipsilateral masseter and a slight topographical effect, without altering the performance of the masticatory motor system significantly. This could represent a feature of the high resilience and adaptability of the stomatognathic system in acute painful conditions.

## Data Availability

Raw data for this study is large and hence can be made available via the corresponding author.
